# Functional connectivity study on visually evoked auditory response based on high-density electroencephalography

**DOI:** 10.3389/fnins.2026.1691902

**Published:** 2026-01-26

**Authors:** Ning Jia, Yueting Feng, Kun Han

**Affiliations:** 1 Department of Neurology, Third Affiliated Hospital of Jinzhou Medical University, Jinzhou, Liaoning, China; 2 Department of Critical Care Medicine, Yantai Yuhuangding Hospital, Yantai, Shandong, China; 3 Department of Neurology, Yingkou Central Hospital, Yingkou, Liaoning, China

**Keywords:** brain activity, EEG, sLORETA, synesthesia, visually evoked auditory response

## Abstract

**Aim:**

This study aimed to investigate the brain activity involved in visually evoked auditory response (vEAR) using high-density electroencephalography (EEG) and explore the differences in connections between visual and auditory cortex.

**Methods:**

Thirty-seven subjects with vEAR and Thirty four subjects without vEAR, matched by age and gender, were recruited. The hearing threshold, years of education, and the Trail Making Test (versions A and B) results were collected from all patients. All participants underwent a 256-channel EEG, and neurophysiological differences were evaluated using standardized low-resolution brain electromagnetic tomography (sLORETA).

**Results:**

Trail Making Test scores in vEAR group were 17.3 ± 2.70 s and 26.28 ± 3.83 s for versions A and B, respectively, and 20.13 ± 6.88 s and 46.65 ± 5.971 s, respectively, in non-vEAR group. Significant difference in version B score was observed between two groups. Compared with non-vEAR group, significant differences were observed at the delta (*p* = 0.005), theta (*p* = 0.016), alpha1 (*p* = 0.016), alpha2 (*p* = 0.011), beta3 (*p* = 0.024), and gamma (*p* = 0.048) frequency bands in vEAR group. In addition, vEAR group showed significantly reduced activation of the posterior cingulate cortex (BA31, *p* = 0.0306) at the alpha2 frequency band and the insular cortex (BA13, *p* = 0.0306) at the beta2 frequency band. Moreover, significantly increased synchronized beta3 connectivity was found between the right part of the cingulate cortex (BA30) and the right primary auditory cortex (BA41) in vEAR group (*p* = 0.045).

**Conclusion:**

vEAR group showed stronger regional connection characteristics than non-vEAR group, which may represent a neural signature associated with vEAR.

## Introduction

Synesthesia is defined as a sensory or cognitive inducer that elicits concurrent sensory sensations that are not experienced by most people under comparable conditions (Ward, 2019). Synesthesia has many forms, the most well-known of which is colors evoked by letters or words, known as grapheme–color synesthesia (G-C synesthesia). The prevalence of synesthesia is about 0.05%, with higher susceptibility among children and women (Rinaldi et al., 2020). A less-known form of synesthesia is visually evoked auditory response (vEAR), which is also referred to as hearing-motion. The prevalence of vEAR may be around 5–20%, making it the most prevalent form of synesthesia (Fassnidge and Freeman, 2018).

Two popular theories about the neural underpinnings of synesthesia are the cross-activation theory and the disinhibition theory (Hubbard, 2007). The cross-activation theory states that synaesthetic precepts result from overabundant connections between brain regions resulting in greater cross-activation of one sensory representation by another (Tilot et al., 2020). Neuroimaging studies of subjects with G-C synesthesia have shown stronger co-activation between the areas responsible for color processing (V4) and the grapheme representation visual word form area, with a spatial proximity between the two areas suggesting that direct cross-activation is the mechanism responsible for this model (Rouw and Scholte, 2007). The disinhibition theory postulates that while there is normal cross-connectivity, the connections are disinhibited in synaesthetes and inhibited in non-synaesthetes, resulting in greater cross-activation. The disinhibited feedback model proposes unusual activation of the concurrently associated brain areas caused by the disinhibition of feedback to these areas from a multisensory nexus area (e.g., the parietal cortex) (Schreiter et al., 2019). However, growing evidence from several neuroimaging studies supports the additional involvement of the parietal cortex in grapheme-color synesthesia, and a combined model of cross-activation together with parietal hyperbinding has been presented as an adequate explanation for synesthetic perception (Lacey et al., 2016).

Most evidence for vEAR has been provided by electroencephalography (EEG) or functional magnetic resonance imaging (fMRI) studies focused on neuroanatomical alterations or effective connectivity analyses (Hubbard, 2007; van Leeuwen et al., 2015). A previous EEG-based study established that hearing-motion arises from early visual processing of motion-related signals and draws on some cross-modal correspondences. Subjects who experience vEAR show a higher amplitude motion-evoked N2 (165–185 ms), and group differences occur at as early as 55–75 ms. However, given the low spatial resolution of EEG, whether the electrophysiological components originated from the visual cortex, auditory cortex, or elsewhere could not be determined (Rothen et al., 2017). Transcranial alternating current stimulation in subjects with vEAR and non-vEAR showed that for subjects in the non-vEAR group, there was a competitive interaction between the visual and auditory areas of the brain, which would usually inhibit each other. However, for the vEAR group, these competitive interactions were largely absent, indicating that the auditory and visual areas of the brain do not compete with each other, but rather cooperate (Fassnidge et al., 2019). More evidence concerning the functional connections involving the cortex underlying vEAR remains to be substantiated using direct physiological measures (e.g., EEG or fMRI) (Fassnidge et al., 2019; Deroy and Spence, 2016; Rothen and Meier, 2014).

Current review holds that vEAR involves the expansion of three cognitive functions: (1) sensory processes (activation of corresponding sensory areas) (Neufeld et al., 2012); (2) a type of cognitive function related to integrating information (the most likely candidate being the parietal lobe) (Braga et al., 2017) and (3) cognitive control processes (related to frontal and parietal cortices) (Rouw et al., 2011; Lalwani and Brang, 2019). It is well known that seeing a person’s lip movements while speaking can enhance speech comprehension and even change our interpretation of speech sounds (Tilot et al., 2020). Visual perception can not only induce the perception of hearing but can also induce new auditory perceptions. There is also evidence that a competitive interaction may exist between the visual and auditory areas of the brain through which they inhibit each other, and that this interaction may be competitive or even cooperative in vEAR (Fassnidge et al., 2019; Sams et al., 1991). The Trail Making Test (TMT) is used as an indicator of visual scanning, graphomotor speed, and executive function (Finney et al., 2001; Dahmen et al., 2017).

High-density EEG is an emerging brain imaging technique that can be used to investigate fast dynamics of electrical activity in the human brain and has been identified as a powerful tool that can yield data with a high temporal resolution and reasonable spatial resolution (Llinas-Regla et al., 2017). High-density EEG enables the non-invasive reconstruction of a region of interest (ROI) by performing source localization analysis methods to scalp-recorded neuronal activities (Toscano et al., 2019). The current research on synesthesia suggests that the activity of the multisensory nexus concurrent-related brain areas is involved in the generation of synesthesia (Ward, 2019). High-density EEG is potentially suited to multimodal forms of the cerebral cortex, and overcomes low spatial resolution of ordinary EEG (Klamer et al., 2015; Dan et al., 2015).

Therefore, in this study we aimed to determine the spectrum of visual and auditory cortex involvement, including the higher cortex, which may be the key to understanding the mechanism of vEAR. Moreover, we used standardized low-resolution brain electromagnetic tomography (sLORETA) source-localized EEG recordings to explore potential relationships between vEAR and control groups in terms of neurophysiological differences.

## Materials and methods

### Participants

Participants were recruited and were shown dynamic pictures that could stimulate sound. Those who could hear the sound for as long as they stared at the picture were included in the vEAR group (37 subjects, 17 men and 20women) with a mean age of 28.5 ± 8.2 years. The matched subjects (15 men and 19 women) with a mean age of 24.1 ± 3.1 years without vEAR constituted the non-vEAR group. The hearing thresholds were collected to ensure that all subjects’ hearing was within the normal range. All participants had normal or corrected-to-normal vision and underwent a personal health screening to ensure that none had previously been diagnosed with any psychiatric disorders. Furthermore, none were taking regular medications. This study was approved by Ethics Committee and all participants provided written informed consent.

### Task and procedure

Seventy one participants were selected and tested in a quiet and well-illuminated room maintained at room temperature. First, the TMT was administered. There are two versions of the TMT (TMT-A and TMT-B). In test A, numbers from 1 to 25 (which are not randomly distributed) are presented to the participants, and they need to connect the numbers in consecutive order from 1 to 25. Test B includes the numbers 1 through 13 and the letters A through L. The participant is required to connect the numbers and letters in consecutive order (1 connected to A, A connected to 2, 2 connected to B, etc.). The subjects were asked to complete the connections as quickly as possible.

For the high-density EEG, the participants were asked not to drink alcohol or caffeinated beverages within 24 h to avoid affecting the results. The participants were asked to wash their hair, wear the EEG electrode cap, and sit comfortably and relax on a chair in a soundproof room for the high-density EEG recordings. A table was placed in front of the chair at a distance of about 30 cm. All subjects sat comfortably in the chair for 5 min, remaining awake and minimizing blinking. During the EEG recording, all participants were instructed to passively view a series of silent, looping GIF animations presented on a computer screen. The stimuli were identical for both groups. The vEAR group was comprised of individuals who self-reported a consistent tendency to experience auditory sensations when viewing such dynamic visual stimuli, whereas the non-vEAR group comprised individuals who reported no such experiences.

### EEG recording and analysis

All participants were asked to sit upright on the chair in a comfortable position. They were asked to refrain from alcohol and caffeinated drinks for 24 h prior to the recording. The EEG was continuously recorded from a 256-channel EG’s HydroCel Geodesic Sensor Net, and Cz was used as the reference channel. Impedances for each electrode were maintained below 50 kΩ. The EEG was sampled at 1000 Hz and amplified 20 times, and the bandpass was filtered between 0.15 and 200 Hz.

The offline EEG analysis was conducted using custom scripts and the EEGLAB toolbox for MATLAB (MathWorks, Natick, MA, USA) (Mikulan et al., 2020). First, the signals for the bandpass were filtered between 0.5 and 70 Hz while using a 50-Hz notch filter. The signals were then resampled at 500 Hz and segmented into 3 s epochs for EEG recording. Subsequently, the electrooculogram and electromyogram artifacts were corrected automatically using the blind source separation-based electrooculogram correction procedure and canonical correlation analysis correction method, respectively, which were available in the automatic artifact removal plug-in (Delorme et al., 2004). Bad channels were identified and interpolated using a spherical spline algorithm. Continuous data were then re-referenced to the average reference. Ocular and cardiac artifacts were removed using an Independent Component Analysis (ICA). Components correlating with stereotypical artifact patterns were visually identified and subtracted from the data.

### Scalp EEG power calculation

For all participants, Welch method of the Hamming window was used and computations were performed using the Spectopo function provided by EEGLAB with 10*log_10_(V2/Hz) to express the power spectrum density.

The frequency bands we focused on were alpha1 (8–10 Hz), alpha2 (10–12 Hz), beta1 (13–18 Hz), beta2 (18.5–21 Hz), beta3 (21.5–30 Hz), delta (2–3.5 Hz), theta (4–7.5 Hz), and gamma (30.5–44 Hz). The relative power of each frequency band was expressed as the average power of each band divided by the average power of 2–45 Hz to eliminate the influence of the brain’s anatomical and neurophysiological characteristics, cranial bone structure, and electrode impedances.

### Standardized low-resolution brain electromagnetic tomography

For source localization, standardized low-resolution brain electromagnetic tomography (sLORETA) was applied. This algorithm provides a single linear solution for the inverse problem without localization bias. The validity of the sources estimated using the sLORETA analysis has been supported by evidence from fMRI and EEG/transcranial magnetic stimulation studies. For this study, the method recommended by the KEY-LORETA software (publicly available for free at: http://www.uzh.ch/keyinst/loreta.htm) developers was used to estimate the locations of the sources of the electrical potentials recorded on the scalp EEG. The sLORETA images (partitioned into 6,239 voxels at 5 mm spatial resolution) from the vEAR group were contrasted with those from the non-vEAR group. It is important to note that, in the absence of individual magnetic resonance imaging (MRI) data, the sLORETA solutions provide low-resolution estimations of source activity based on a standardized head model. The referenced Brodmann areas (BA) serve as approximate anatomical labels.

### Functional connectivity

The linear connectivity was calculated as described previously (Gomez-Herrero et al., 2006). Based on previous studies, three bilateral ROIs were defined: (1) the visual cortex (BA17, BA18, BA19, BA20, BA21, BA22). (2) the posterior cingulate cortex (PCC) (BA 30, BA31, BA32), and (3) the auditory cortex (BA41, BA42) (Alonso-Montes et al., 2015; van Kerkoerle et al., 2017; Glickfeld and Olsen, 2017; Leech and Smallwood, 2019; Newson and Thiagarajan, 2019).

### Statistical analysis

Descriptive data are expressed as the mean ± standard deviation. The proportion of men and women between the groups were compared using the chi-square test, and a one-way analysis of variance was used to compare the ages. The TMT scores between the vEAR and the non-vEAR group were measured using the Mann–Whitney U test (median, interquartile ranges). For all analyses, a *p*-value < 0.05 was considered statistically significant. All data analyses were performed using IBM SPSS Statistics v. 23 (SPSS/PC, Chicago, IL, USA). For the functional connectivity analysis, statistical significance of connections between all predefined ROI pairs across frequency bands was assessed. To control for the multiple comparisons inherent in such an analysis, the False Discovery Rate (FDR) correction was applied. Only connections surviving an FDR-corrected *p* value < 0.05 were reported as significant.

## Results

### Patient and demographic characteristics

**Figure 1:**
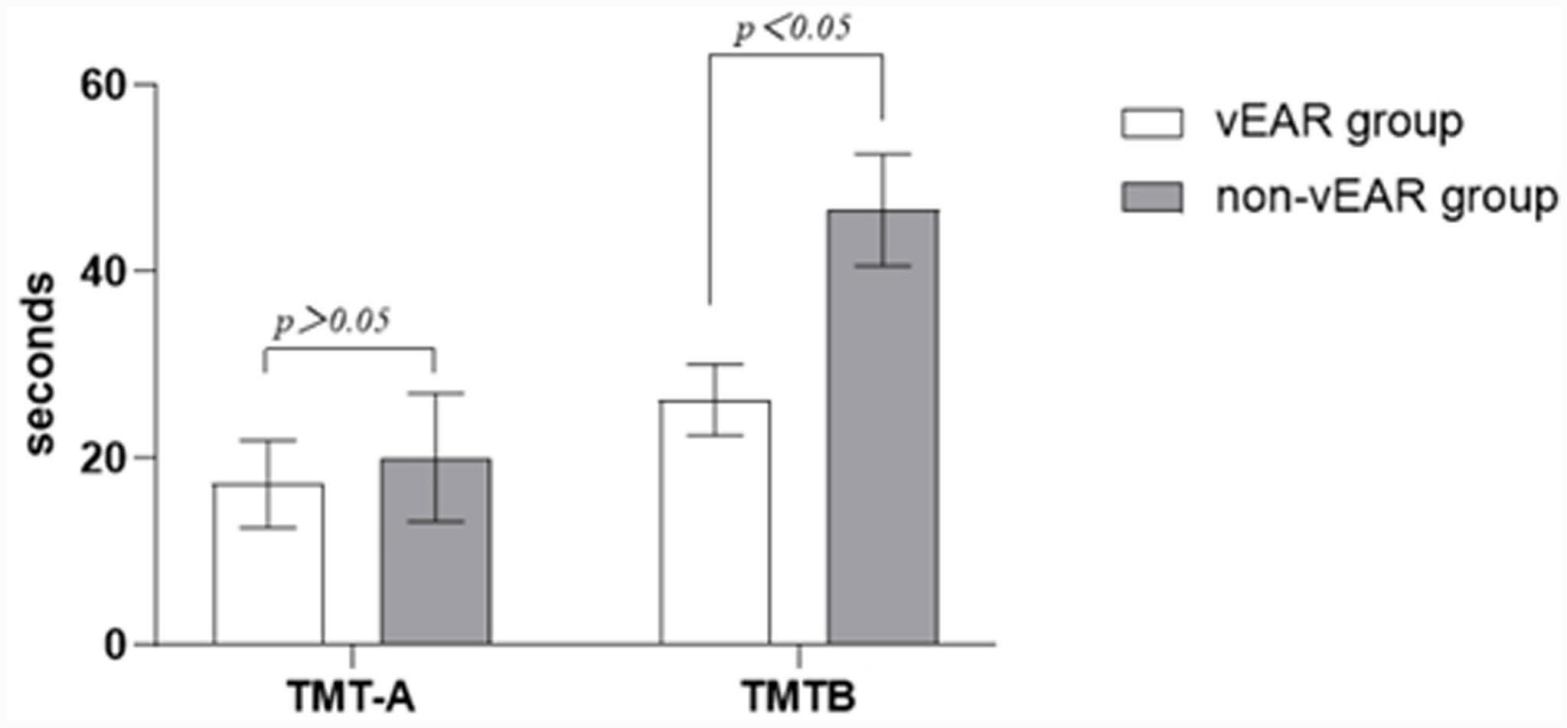
Comparison of TMT results between vEAR and non-vEAR groups. VEar group had a significantly lower TMT-B score than non-vEAR group. TMT, trial making test; vEAR, visually evoked auditory response.

The TMT-A score for the vEAR group was 17.3 ± 2.7 s, while that for the non-vEAR group was 20.0 ± 6.9 s, and the difference between the two groups was not significant. There was also no difference in education level between the two groups. The TMT-B score for the vEAR group was 26.3 ± 3.8 s, while that for the non-vEAR group was 46.7 ± 6.0 s, with a significant difference between the two groups (Table 1). The vEAR group completed part B of the TMT significantly faster than the non-vEAR group (Figure 1).

**Table 1:** Patient characteristics of the vEAR and the non-vEAR groups.

Parameters	vEAR group	non-vEAR group	*p* value
Age (Y)	28.5 ± 8.1	24.1 ± 3.3	0.429
Sex (men/women)	17/20	15/19	0.497
Years of education (Y)	18 ± 3.2	16 ± 0.0	0.329
Hearing threshold (dB)	2.5 ± 5	4 ± 8.7	0.441
TMT-A	17.3 ± 2.7	20.0 ± 6.9	0.396
TMT-B	26.3 ± 3.8	46.7 ± 6.0	0.005*

TMT-A, Trail Making Test-A; TMT-B, Trail Making Test-B; vEAR: visually-evoked auditory response. *p < 0.05 for differences between two groups.

### EEG analysis

To assess whether the observed relative power differences were driven by alterations in specific bands rather than global power shifts, we examined the normalized absolute power (by z-scoring across subjects within each group). The pattern of between-group differences in normalized absolute power remained consistent with the relative power findings described below (Pernet et al., 2015; Rolls, 2019).

**Figure 2:**
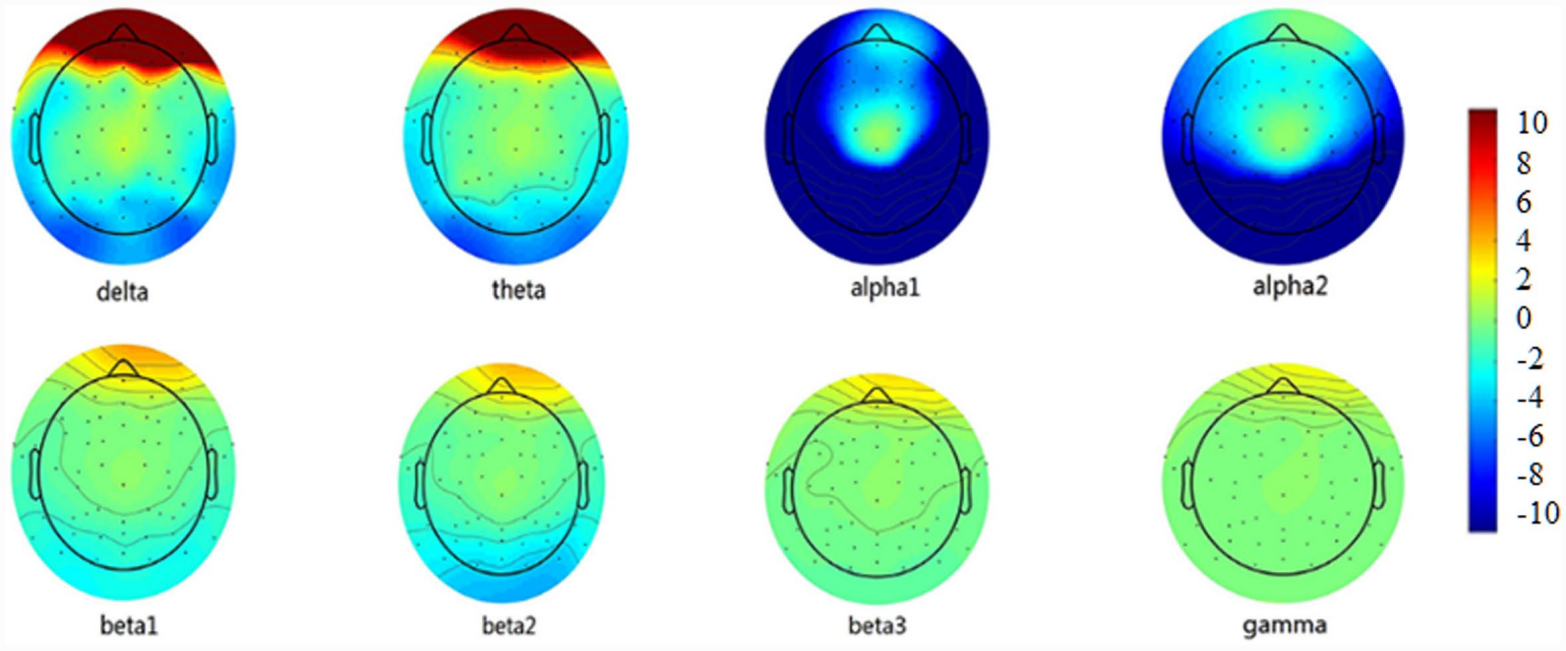
Comparison of the average EEG power at eight frequency bands between vEAR and non-vEAR group. VEAr group had significantly higher levels of delta and theta frequency bands and relatively lower levels of alpha1, alpha2, beta3, and gamma frequency bands (*p* < 0.05). EEG, electroencephalography; vEAR, visually evoked auditory response.

**Figure 3:**
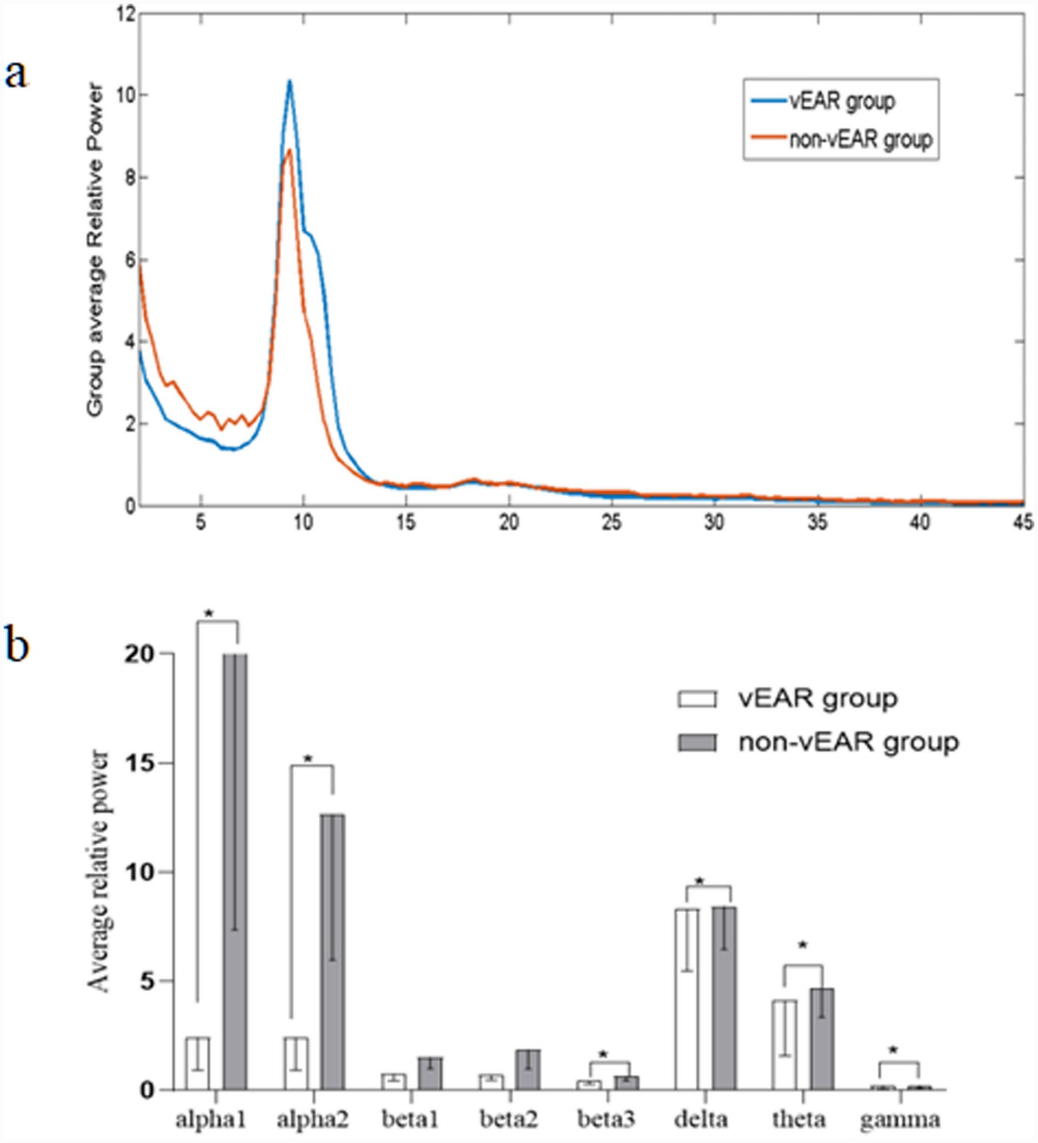
EEG results for vEAR and non-vEAR group. **(a)** Distribution of average brain activity along the eight frequency bands in each group; the unit for X-axis was Hz. **(b)** Comparison between the average EEG power at eight frequency bands between vEAR and non-vEAR group. *p < 0.05 against non-vEAR group. EEG, electroencephalography; vEAR, visually evoked auditory response.

EEG analysis of vEAR and non-vEAR groups was shown in Figures 2, 3. The power spectrum densities recorded across all electrodes in each group were averaged to show the distribution of brain activity along the frequency bands ranging from delta (2–3.5 Hz) to gamma (30.5–44 Hz). Compared with the non-vEAR group, the vEAR group showed significantly higher levels of delta and theta frequency bands and significantly lower levels of alpha1, alpha2, beta3, and gamma frequency bands (*p* < 0.05).

### Source localization

**Figure 4:**
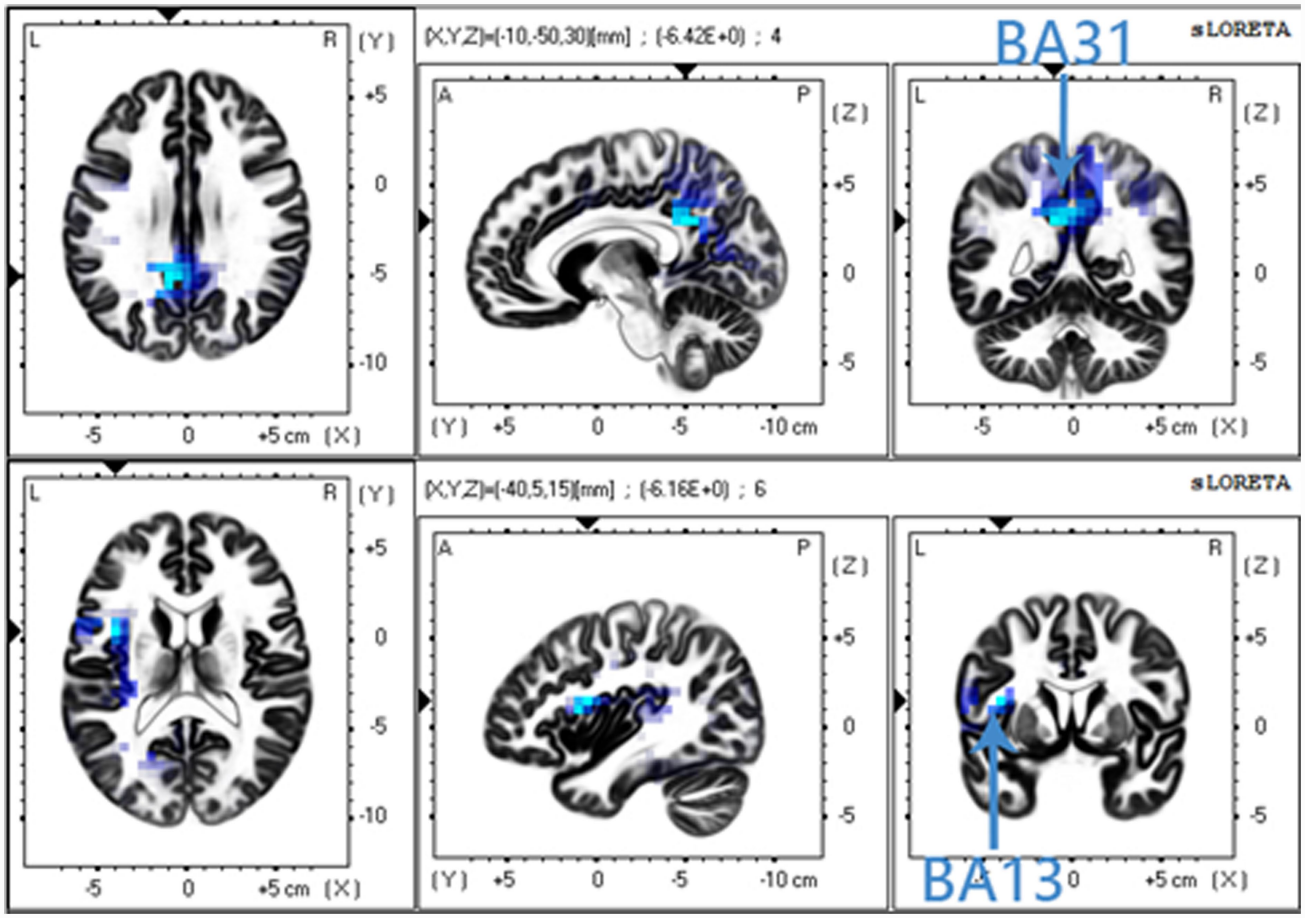
Comparison of sLORETA results between vEAR and non-vEAR group. Compared with non-vEAR group, vEAR group showed less activity in the posterior cingulate cortex (PCC) (BA31) for the alpha2 frequency band and less activity in the insular cortex (BA13) for the beta-2 frequency band. sLORETA, standardized low-resolution brain electromagnetic tomography; vEAR, visually evoked auditory response.

Compared with the non-vEAR group, the vEAR group showed significantly less activity in the PCC (BA31) for the alpha2 frequency band and in the insular cortex (BA13) for the beta2 frequency band (Figure 4). The PCC (BA31) is mainly associated with visual imagination and situational memory retrieval, and the insular cortex (BA13) processes sensory experiences generated by convergence information.

### Functional connectivity

**Figure 5:**
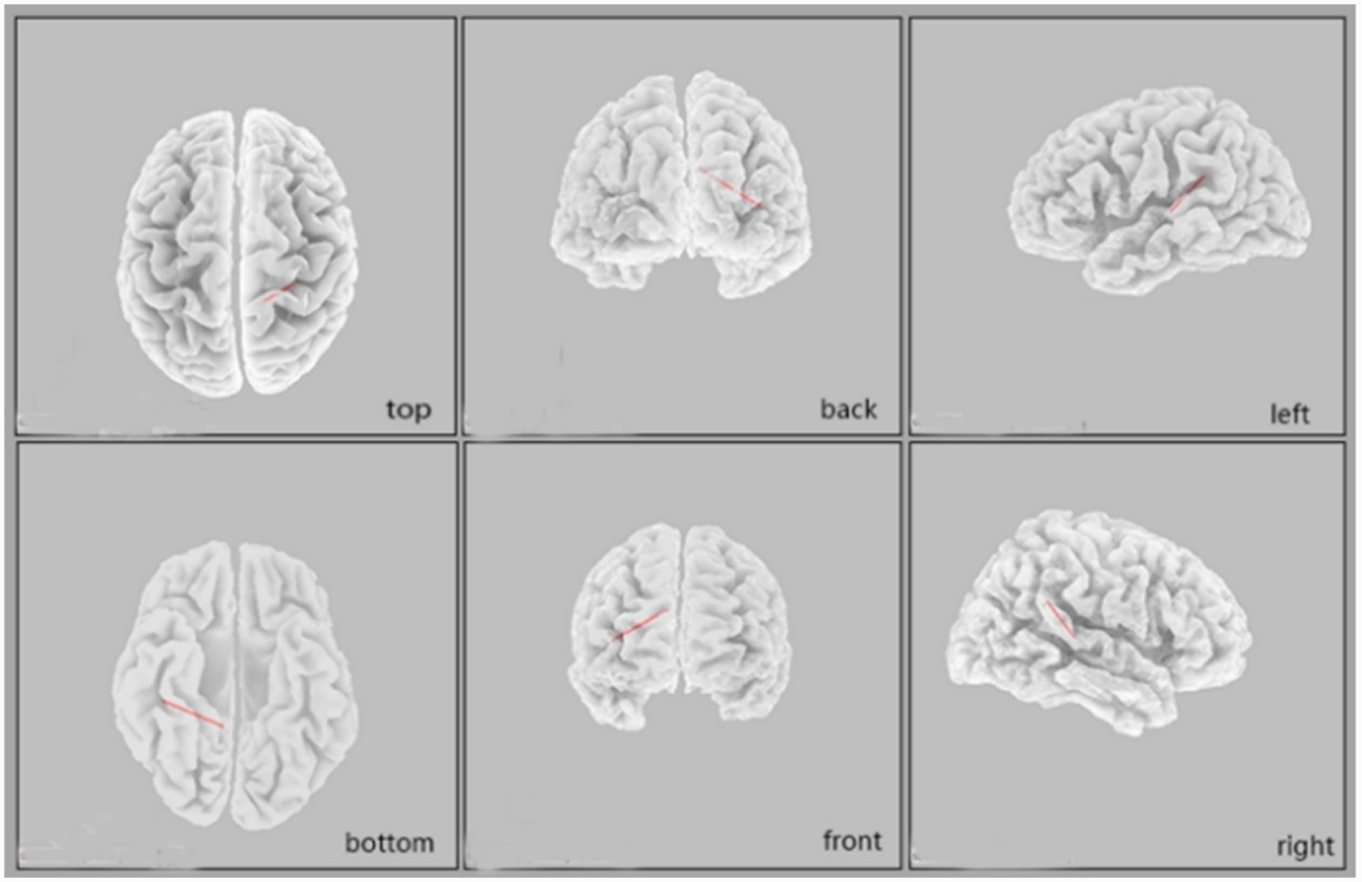
Comparison of functional connectivity between vEAR and non-vEAR group in sLORETA source space. Higher beta-3 linear connectivity between the right BA30 and the right BA41 was observed in vEAR group compared to non-vEAR group. sLORETA, standardized low-resolution brain electromagnetic tomography; vEAR, visually evoked auditory response.

After applying False Discovery Rate (FDR) correction for multiple comparisons across all ROI pairs and frequency bands, a significant difference in functional connectivity was observed in the beta3 frequency band (corrected *p* = 0.045). Specifically, the vEAR group showed significantly higher lagged phase synchronization (i.e., functional connectivity) between the right cingulate cortex (BA30) and the right primary auditory cortex (BA41) in the beta3 frequency band compared to the non-vEAR group (Figure 5).

## Discussion

Current neurophysiological models suggest that synesthesia is associated with a hyperconnected and hyperactivated brain, and synesthesia might be a consequence of globally altered brain network connectivity (Hackett, 2015; Grossenbacher and Lovelace, 2001; Asano et al., 2019). In this study, we explored the characteristics of neural activity in subjects with vEAR. The TMT-B score was lower in vEAR group than in non-vEAR group, indicating a significantly better performance in vEAR group. EEG results also showed significant differences between vEAR and non-vEAR groups. Compared with non-vEAR group, vEAR group had significantly higher levels of delta and theta frequency bands and significantly lower levels of alpha-1, alpha2, beta3, and gamma frequency bands. We further used sLORETA to identify the dominant brain areas associated with these differences in EEG power and found that vEAR group showed lower activity in the PCC (BA31) for the alpha2 frequency band and lower activity in the insular cortex (BA13) for the beta2 frequency band compared with non-vEAR group. Functional connectivity analysis revealed increased beta3 linear connectivity between the right cingulate cortex (BA30) and the right primary auditory cortex (BA41) in the vEAR group.

The superior performance of the vEAR group on the TMT-B suggests a potential association between vEAR propensity and efficiency in certain cognitive operations involving task-switching and visual-motor tracking, although confounding factors such as subtle differences in age or cognitive strategy cannot be excluded. The significant difference in TMT-B scores in our study indicates that the subjects in vEAR group had better working memory and inhibition control than those in non-vEAR group, consistent with previous reports that synesthesia is associated with “positive” changes in cognitive function (Hackett, 2015). It has been reported that subjects with G-C and other forms of synesthesia perform better on cognitive processes and memory (Grossenbacher and Lovelace, 2001; Proverbio et al., 2011). Since multiple cognitive functions are involved in the TMT test, task performance activates the right inferior medial frontal cortex as well as other non-frontal brain regions and the parietal sulcus (Braga et al., 2017). It is possible that in subjects with synesthesia, more sensory channels are activated, more areas of the brain are involved, and the related brain areas are more efficient (Chan et al., 2015).

The observed spectral power differences may be linked to distinct cognitive processes. Rhythmic electrical activity in discrete frequency bands in vEAR accompanies a broad range of motor, affective, and cognitive processes in the brain (Adams et al., 2019). There is evidence showing that theta and delta frequency band dynamics dominate the spectral profile of cortical activity during semantic processing (Adams et al., 2019). In addition, lower theta frequency may coordinate the integration of hippocampal contextual information (Remondes and Wilson, 2013). In this study, vEAR group showed significantly higher levels of delta and theta frequency bands, and lower levels of alpha1, alpha2, beta3, and gamma frequency bands compared with non-vEAR group. Alpha rhythms control access to stored memories, and beta rhythm generation correlates with task performance, requiring short-term memory and prediction (Başar et al., 2001; Klimesch, 2012). Gamma rhythms organize primary sensory information to facilitate higher-order processing (Arnal and Giraud, 2012). Although it has been shown that synaesthetes have better cognition, memory, and other functions than non-synaesthetes, the mutual influence of cortical frequency band activity still needs to be studied. Therefore, understanding theta and delta frequency band dynamics is essential for determining the processing of multiple senses in vEAR group.

Resting-state network measurements show that, similar to other synesthesias, parietal brain regions are involved in vEAR (Fries, 2015; Barnett et al., 2008), which suggests that the parietal lobe may be a key brain area for vEAR. The sLORETA analysis showed that, compared with non-vEAR group, vEAR group had lower activity in the left upper PCC (BA31), which is mainly associated with visual imagination and situational memory retrieval in the alpha2 frequency band. vEAR group had lower activation of BA13, which is located in the insular cortex and processes sensory experiences generated by convergence information. A review focused on synaesthetic brain areas showed that many synaesthetic patterns involve activation clusters in the parietal lobe; thus, this brain region has gained increasing attention in the field of synesthesia research (Rouw et al., 2011; Rothen et al., 2017). The frontoparietal network is often implicated in both auditory and visual information processing (Fries, 2015); however, it is unclear whether the same frontoparietal regions are recruited for both auditory and visual tasks. The evidence suggests that the location of frontoparietal activation during a cognitive task is likely to be affected by intrinsic biases toward auditory or visual inputs for speech production (Proverbio et al., 2011; Caron-Desrochers et al., 2018). Generally, comprehension is typically regarded as a function of ventral prefrontal regions such as Broca’s area, while eye movement and saccade planning are localized in the dorsal frontoparietal cortices (Goller et al., 2009). Insula activation could be related to the conversion process of a particular external stimulus to an internal stimulus, and activation in this region might also be related to the emotional quality that often accompanies synaesthetic experiences, as synaesthetes often associate a particular feeling with the synaesthetic experience (Rouw and Root, 2019; Simner et al., 2005). Although our results show less change in parietal lobe activity in vEAR group, increases or reductions in cortical activity differ across types of synesthesia.

The increased linear connectivity in the beta3 frequency band between the cingulate cortex (BA30) and the right primary auditory cortex (BA41) may suggest their association with the occurrence of vEAR. A part of the anterior cuneiform and the posterior cingulate gyrus are used for visual space imaging, episodic memory retrieval, and proficient recognition (Newson and Thiagarajan, 2019). Furthermore, the involvement of the right auditory cortex as a strong hub in auditory–visual synesthesia was recently demonstrated by resting-state EEG connectivity analysis (Freeman, 2020). A study using fMRI found a stronger connectivity of the left inferior parietal cortex with the left primary auditory and right primary visual cortex in a group of auditory–visual synaesthetes compared with controls (Brauchli et al., 2018). The current G-C synesthesia review showed that V4 is an important, but not sufficient, component associated with synaesthetic color experiences (Goller et al., 2009). Higher beta3 linear connectivity between an area of the cingulate cortex (BA30) and the right primary auditory cortex (BA41) was observed in the vEAR group relative to the non-vEAR group in our study. This finding suggests that vEAR depends on the disinhibition of normally occurring latent cross-connectivity between the visual and auditory cortices (Niccolai et al., 2012). Therefore, the right auditory cortex may act as a concurrent representation area, but it is not the only visual area involved in vEAR processing.

Although synesthesia is often referred to as a neurological condition and there is a higher detection rate in patients with mental disorders, it is not listed in the DSM IV or the ICD classifications since it generally does not interfere with normal daily functioning; therefore, there are no clear guidelines for the diagnosis of synesthesia (Krugliak and Noppeney, 2016; Nanay, 2018).

This study has several limitations that should be considered when interpreting the results. First, the classification of participants into vEAR and non-vEAR groups was based on self-report without quantitative assessment of the percept’s vividness, frequency, or temporal dynamics during the EEG session (Fassnidge and Freeman, 2018). Therefore, the observed neural differences are best interpreted as traits associated with individuals prone to vEAR, rather than direct neural correlates of the ongoing illusory experience itself. Second, the experimental paradigm involved passive viewing without time-locked behavioral responses, which limits our ability to dissect brain activity specifically related to vEAR moments from general visual processing and attentional states. Furthermore, we did not exclude the possibility that vEAR participants might also have other forms of synesthesia, an under-appreciated variable that could influence the findings (Shams et al., 2005; Arend et al., 2020; Asher et al., 2009). Additionally, due to the design of the study, the directionality of the observed functional connections cannot be inferred. Finally, the sample size was limited to 37 vEAR participants, and future studies with larger samples and more precise diagnostic evidence are needed to further characterize synesthetes. Furthermore, methodological constraints precluded the analysis of absolute power. Although we validated our relative power findings with an alternative normalization approach and the pattern of results was consistent, future studies should include direct measures of absolute power to provide complete electrophysiological characterization and rule out any potential normalization artifacts. Future studies should incorporate real-time perceptual reporting (e.g., button presses) and validated psychophysical measures to better capture and quantify the vEAR experience (Rothen et al., 2017; Fassnidge et al., 2019). Such approaches would help establish reliable neural distinctions between synesthetes and non-synesthetes.

In conclusion, our study showed that people with vEAR performed better in the processing of visual or auditory tasks, as shown by the TMT-B. A significantly higher level of delta and theta frequency bands and relatively lower levels of alpha1, alpha2, beta3, and gamma frequency bands in the vEAR group were also observed. Moreover, vEAR group showed stronger regional connection characteristics than non-vEAR group, which may constitute a neurophysiological correlate of vEAR. HD-EEG technology has significant advantages for detecting changes in brain function in subjects with vEAR, and our results based on HD-EEG may have generalizable implications for understanding the mechanisms underlying individual differences among subjects with vEAR.

## Data Availability

The original contributions presented in the study are included in the article/supplementary material, further inquiries can be directed to the corresponding author.

## References

[ref1] Adams N. E. Teige C. Mollo G. Karapanagiotidis T. Cornelissen P. L. Smallwood J. . (2019). Theta/delta coupling across cortical laminae contributes to semantic cognition. J. Neurophysiol. 121, 1150–1161. doi: 10.1152/jn.00686.2018, 30699059 PMC6485732

[ref2] Alonso-Montes C. Diez I. Remaki L. Escudero I. Mateos B. Rosseel Y. . (2015). Lagged and instantaneous dynamical influences related to brain structural connectivity. Front. Psychol. 6:1024. doi: 10.3389/fpsyg.2015.01024, 26257682 PMC4508482

[ref3] Arend I. Yuen K. Ashkenazi S. Henik A. (2020). Space counts! Brain correlates of spatial and numerical representations in synaesthesia. Cortex 122, 300–310. doi: 10.1016/j.cortex.2018.11.006, 30527926

[ref4] Arnal L. H. Giraud A. L. (2012). Cortical oscillations and sensory predictions. Trends Cogn. Sci. 16, 390–398. doi: 10.1016/j.tics.2012.05.003, 22682813

[ref5] Asano M. Takahashi S. I. Tsushiro T. Yokosawa K. (2019). Synaesthetic colour associations for Japanese kanji characters: from the perspective of grapheme learning. Philos. Trans. R. Soc. Lond. Ser. B Biol. Sci. 374:20180349. doi: 10.1098/rstb.2018.0349, 31630661 PMC6834007

[ref6] Asher J. E. Lamb J. A. Brocklebank D. Cazier J. B. Maestrini E. Addis L. . (2009). A whole-genome scan and Fine-mapping linkage study of auditory-visual Synesthesia reveals evidence of linkage to chromosomes 2q24, 5q33, 6p12, and 12p12. Am. J. Hum. Genet. 84, 279–285. doi: 10.1016/j.ajhg.2009.01.012, 19200526 PMC2668015

[ref7] Barnett K. J. Foxe J. J. Molholm S. Kelly S. P. Shalgi S. Mitchell K. J. . (2008). Differences in early sensory-perceptual processing in synesthesia: a visual evoked potential study. NeuroImage 43, 605–613. doi: 10.1016/j.neuroimage.2008.07.028, 18723094

[ref8] Başar E. Başar-Eroglu C. Karakaş S. Schürmann M. (2001). Gamma, alpha, delta, and theta oscillations govern cognitive processes. Int. J. Psychophysiol. 39, 241–248. doi: 10.1016/s0167-8760(00)00145-8, 11163901

[ref9] Braga R. M. Hellyer P. J. Wise R. J. Leech R. (2017). Auditory and visual connectivity gradients in frontoparietal cortex. Hum. Brain Mapp. 38, 255–270. doi: 10.1002/hbm.23358. Epub 2016 Aug 29, 27571304 PMC5215394

[ref10] Brauchli C. Elmer S. Rogenmoser L. Burkhard A. Jäncke L. (2018). Top-down signal transmission and global hyperconnectivity in auditory-visual synesthesia: evidence from a functional EEG resting-state study. Hum. Brain Mapp. 39, 522–531. doi: 10.1002/hbm.23861, 29086468 PMC6866463

[ref11] Caron-Desrochers L. Schonwiesner M. Focke K. Lehmann A. (2018). Assessing visual modulation along the human subcortical auditory pathway. Neurosci. Lett. 685, 12–17. doi: 10.1016/j.neulet.2018.07.020, 30009874

[ref12] Chan E. MacPherson S. E. Robinson G. Turner M. Lecce F. Shallice T. . (2015). Limitations of the trail making test part-B in assessing frontal executive dysfunction. J. Int. Neuropsychol. Soc. 21, 169–174. doi: 10.1017/S135561771500003X, 25697352

[ref13] Dahmen J. Cook D. Fellows R. Schmitter-Edgecombe M. (2017). An analysis of a digital variant of the trail making test using machine learning techniques. Technol. Health Care 25, 251–264. doi: 10.3233/THC-161274 PMC5384876 27886019

[ref14] Dan B. Pelc K. Cebolla A. M. Cheron G. (2015). High-density electroencephalography developmental neurophysiological trajectories. Dev. Med. Child Neurol. 57, 44–47. doi: 10.1111/dmcn.12728 25800492

[ref15] Delorme A. Makeig S. Delorme A. Makeig S. (2004). EEGLAB: an open source toolbox for analysis of single-trial EEG dynamics including independent component analysis. J. Neurosci. Methods 134, 9–21. doi: 10.1016/j.jneumeth.2003.10.009, 15102499

[ref16] Deroy O. Spence C. (2016). Lessons of synaesthesia for consciousness: learning from the exception, rather than the general. Neuropsychologia 88, 49–57. doi: 10.1016/j.neuropsychologia.2015.08.005, 26290957

[ref17] Fassnidge C. Ball D. Kazaz Z. Knudsen S. Spicer A. Tipple A. . (2019). Hearing through your eyes: neural basis of audiovisual cross-activation, revealed by transcranial alternating current stimulation. J. Cogn. Neurosci. 31, 922–935. doi: 10.1162/jocn_a_01395, 30883286

[ref18] Fassnidge C. J. Freeman E. D. (2018). Sounds from seeing silent motion: who hears them, and what looks loudest? Cortex 103, 130–141. doi: 10.1016/j.cortex.2018.02.019, 29625386

[ref19] Finney E. M. Fine I. Dobkins K. R. (2001). Visual stimuli activate auditory cortex in the deaf. Nat. Neurosci. 4, 1171–1173. doi: 10.1038/nn763 11704763

[ref20] Freeman E. D. (2020). Hearing what you see: distinct excitatory and disinhibitory mechanisms contribute to visually-evoked auditory sensations. Cortex 131, 66–78. doi: 10.1016/j.cortex.2020.06.014, 32801076

[ref21] Fries P. (2015). Rhythms for cognition: communication through coherence. Neuron 88, 220–235. doi: 10.1016/j.neuron.2015.09.034, 26447583 PMC4605134

[ref22] Glickfeld L. L. Olsen S. R. (2017). Higher-order areas of the mouse visual cortex. Annu. Rev. Vis. Sci. 3, 251–273. doi: 10.1146/annurev-vision-102016-061331, 28746815

[ref23] Goller A. I. Otten L. J. Ward J. (2009). Seeing sounds and hearing colors: an event-related potential study of auditory-visual synesthesia. J Cogn Neuro 21, 1869–1881. doi: 10.1162/jocn.2009.21134, 18823243

[ref24] Gomez-Herrero G. Clercq W. Anwar H. Kara O. Egiazarian K. Van Huffel S. (2006). Automatic removal of ocular artifacts in the EEG without an EOG Reference Channel. Proceedings of the 7th Nordic Signal Processing Symposium - NORSIG 2006. 130–133.

[ref25] Grossenbacher P. G. Lovelace C. T. (2001). Mechanisms of synesthesia: cognitive and physiological constraints. Trends Cogn. Sci. 5, 36–41. doi: 10.1016/s1364-6613(00)01571-0, 11164734

[ref26] Hackett T. A. (2015). Anatomic organization of the auditory cortex. Handb. Clin. Neurol. 129, 27–53. doi: 10.1016/B978-0-444-62630-1.00002-0 25726261

[ref27] Hubbard E. M. (2007). Neurophysiology of synesthesia. Curr. Psychiatry Rep. 9, 193–199. doi: 10.1007/s11920-007-0018-6, 17521514

[ref28] Klamer S. Elshahabi A. Lerche H. Braun C. Erb M. Scheffler K. . (2015). Differences between MEG and High-density EEG source localizations using a distributed source model in comparison to fMRI. Brain Topogr. 28, 87–94. doi: 10.1007/s10548-014-0405-3, 25296614

[ref29] Klimesch W. (2012). Α-Band oscillations, attention, and controlled access to stored information. Trends Cogn. Sci. 16, 606–617. doi: 10.1016/j.tics.2012.10.007, 23141428 PMC3507158

[ref30] Krugliak A. Noppeney U. (2016). Synaesthetic interactions across vision and audition. Neuropsychologia 88, 65–73. doi: 10.1016/j.neuropsychologia.2015.09.027, 26427739

[ref31] Lacey S. Martinez M. McCormick K. Sathian K. (2016). Synesthesia strengthens sound-symbolic cross-modal correspondences. Eur. J. Neurosci. 44, 2716–2721. doi: 10.1111/ejn.13381, 27564319 PMC5089906

[ref32] Lalwani P. Brang D. (2019). Stochastic resonance model of synaesthesia. Philos. Trans. R. Soc. Lond. Ser. B Biol. Sci. 374:20190029. doi: 10.1098/rstb.2019.0029, 31630652 PMC6834013

[ref33] Leech R. Smallwood J. (2019). The posterior cingulate cortex: insights from structure and function. Handb. Clin. Neurol. 166, 73–85. doi: 10.1016/B978-0-444-64196-0.00005-4, 31731926

[ref35] Llinas-Regla J. Vilalta-Franch J. Lopez-Pousa S. Calvó-Perxas L. Rodas D. T. Garre-Olmo J. (2017). The trail making test. Assessment 24, 183–196. doi: 10.1177/1073191115602552 26318386

[ref36] Mikulan E. Russo S. Parmigiani S. Sarasso S. Zauli F. M. Rubino A. . (2020). Simultaneous human intracerebral stimulation and HD-EEG, ground-truth for source localization methods. Sci Data 7:127. doi: 10.1038/s41597-020-0467-x, 32345974 PMC7189230

[ref37] Nanay B. (2018). Multimodal mental imagery. Cortex 105, 125–134. doi: 10.1016/j.cortex.2017.07.006, 28801065 PMC6079145

[ref38] Neufeld J. Sinke C. Zedler M. Dillo W. Emrich H. M. Bleich S. . (2012). Disinhibited feedback as a cause of synesthesia: evidence from a functional connectivity study on auditory-visual synesthetes. Neuropsychologia 50, 1471–1477. doi: 10.1016/j.neuropsychologia.2012.02.032, 22414594

[ref39] Newson J. J. Thiagarajan T. C. (2019). EEG frequency bands in psychiatric disorders: a review of resting state studies. Front. Hum. Neurosci. 12:521. doi: 10.3389/fnhum.2018.00521, 30687041 PMC6333694

[ref40] Niccolai V. van Leeuwen T. M. Blakemore C. Stoerig P. (2012). Synaesthetic perception of colour and visual space in a blind subject: an fMRI case study. Conscious. Cogn. 21, 889–899. doi: 10.1016/j.concog.2012.03.010, 22507663

[ref41] Pernet C. R. Latinus M. Nichols T. E. Rousselet G. A. (2015). Cluster-based computational methods for mass univariate analyses of event-related brain potentials/fields: a simulation study. J. Neurosci. Methods 250, 85–93. doi: 10.1016/j.jneumeth.2014.08.003, 25128255 PMC4510917

[ref42] Proverbio A. M. D'Aniello G. E. Adorni R. Zani A. (2011). When a photograph can be heard: vision activates the auditory cortex within 110 ms. Sci. Rep. 1:54. doi: 10.1038/srep00054, 22355573 PMC3216541

[ref43] Remondes M. Wilson M. A. (2013). Cingulate-hippocampus coherence and trajectory coding in a sequential choice task. Neuron 80, 1277–1289. doi: 10.1016/j.neuron.2013.08.037, 24239123 PMC3858450

[ref44] Rinaldi L. J. Smees R. Carmichael D. A. Simner J. (2020). Numeracy skills in child synaesthetes: evidence from grapheme-colour synaesthesia. Cortex 126, 141–152. doi: 10.1016/j.cortex.2020.01.007, 32078819

[ref45] Rolls E. T. (2019). The cingulate cortex and limbic systems for emotion, action, and memory. Brain Struct. Funct. 224, 3001–3018. doi: 10.1007/s00429-019-01945-2, 31451898 PMC6875144

[ref46] Rothen N. Bartl G. Franklin A. Ward J. (2017). Electrophysiological correlates and psychoacoustic characteristics of hearing-motion synaesthesia. Neuropsychologia 106, 280–288. doi: 10.1016/j.neuropsychologia.2017.08.031, 28982544

[ref47] Rothen N. Meier B. (2014). Acquiring synaesthesia: insights from training studies. Front. Hum. Neurosci. 8:109. doi: 10.3389/fnhum.2014.00109 24624072 PMC3939620

[ref48] Rouw R. Root N. B. (2019). Distinct colours in the ‘synaesthetic colour palette’. Philos Trans Royal Soc B 374:20190028. doi: 10.1098/rstb.2019.0028, 31630651 PMC6834014

[ref49] Rouw R. Scholte H. S. (2007). Increased structural connectivity in grapheme-color synesthesia. Nat. Neurosci. 10, 792–797. doi: 10.1038/nn1906, 17515901

[ref50] Rouw R. Scholte H. S. Colizoli O. (2011). Brain areas involved in synaesthesia: a review. J. Neuropsychol. 5, 214–242. doi: 10.1111/j.1748-6653.2011.02006.x, 21923787

[ref51] Sams M. Aulanko R. Hämäläinen M. Hari R. Lounasmaa O. V. Lu S. T. . (1991). Seeing speech: visual information from lip movements modifies activity in the human auditory cortex. Neuroence Lett 127, 141–145. doi: 10.1016/0304-3940(91)90914-f 1881611

[ref52] Schreiter M. L. Chmielewski W. X. Ward J. Beste C. (2019). How non-veridical perception drives actions in healthy humans: evidence from synaesthesia. Philos. Trans. R. Soc. Lond. Ser. B Biol. Sci. 374:20180574. doi: 10.1098/rstb.2018.0574, 31630650 PMC6834016

[ref53] Shams L. Kamitani Y. Thompson S. Shimojo S. (2005). Sound alters visual evoked potentials in humans. Neuroreport 12, 3849–3852. doi: 10.1097/00001756-200112040-00049 11726807

[ref54] Simner J. Ward J. Lanz M. Jansari A. Noonan K. Glover L. . (2005). Non-random associations of graphemes to colours in synaesthetic and non-synaesthetic populations. Cogn. Neuropsychol. 22, 1069–1085. doi: 10.1080/02643290500200122, 21038290

[ref55] Tilot A. K. Vino A. Kucera K. S. Carmichael D. A. van den Heuvel L. den Hoed J. . (2020). Correction to 'investigating genetic links between grapheme-colour synaesthesia and neuropsychiatric traits'. Philos. Trans. R. Soc. Lond. Ser. B Biol. Sci. 375:20190746. doi: 10.1098/rstb.2019.0746, 32075566 PMC7061983

[ref56] Toscano G. Carboni M. Rubega M. Spinelli L. Pittau F. Bartoli A. . (2019). Visual analysis of high density EEG: as good as electrical source imaging? Clin. Neurophysiol. Pract. 5, 16–22. doi: 10.1016/j.cnp.2019.09.002 31909306 PMC6939057

[ref57] van Kerkoerle T. Self M. W. Roelfsema P. R. (2017). Layer-specificity in the effects of attention and working memory on activity in primary visual cortex. Nat. Commun. 8:13804. doi: 10.1038/ncomms13804, 28054544 PMC5227065

[ref58] van Leeuwen T. M. Singer W. Nikolić D. (2015). The merit of Synesthesia for consciousness research. Front. Psychol. 6:1850. doi: 10.3389/fpsyg.2015.01850, 26696921 PMC4667101

[ref59] Ward J. (2019). Synaesthesia: a distinct entity that is an emergent feature of adaptive neurocognitive differences. Philos. Trans. R. Soc. Lond. Ser. B Biol. Sci. 374:20180351. doi: 10.1098/rstb.2018.0351 31630648 PMC6834018

